# Phage Ligands for Identification of Mesenchymal-Like Breast Cancer Cells and Cancer-Associated Fibroblasts

**DOI:** 10.3389/fonc.2018.00625

**Published:** 2018-12-17

**Authors:** Kelvin M. Jones, Balasubramanyam Karanam, Jacqueline Jones-Triche, Maninder Sandey, Henry J. Henderson, Rajeev S. Samant, Samuel Temesgen, Clayton Yates, Deepa Bedi

**Affiliations:** ^1^Department of Biomedical Sciences, College of Veterinary Medicine, Tuskegee University, Tuskegee, AL, United States; ^2^Department of Biology, Center for Cancer Research, Tuskegee University, Tuskegee, AL, United States; ^3^Department of Biology and Environmental Sciences, Troy University, Troy, AL, United States; ^4^Department of Pathobiology, Auburn University, Auburn, AL, United States; ^5^Department of Pathobiology, The University of Alabama at Birmingham, Birmingham, AL, United States; ^6^Department of Pathobiology, College of Veterinary Medicine, Tuskegee University, Tuskegee, AL, United States

**Keywords:** Phage display, breast cancer, fibroblasts, EMT, cancer-associated fibroblasts

## Abstract

Epithelial to mesenchymal transition (EMT) is believed to be crucial for primary tumors to escape their original residence and invade and metastasize. To properly define EMT, there is a need for ligands that can identify this phenomenon in tumor tissue and *invivo*. A phage-display selection screening was performed to select novel binding phage peptides for identification of EMT in breast cancer. Epithelial breast cancer cell line, MCF-7 was transformed to mesenchymal phenotype by TGF-β treatment and was used for selection. Breast fibroblasts were used for subtractive depletion and breast cancer metastatic cell lines MDA-MB-231, T47D-shNMI were used for specificity assay. The binding peptides were identified, and their binding capacities were confirmed by phage capture assay, phage-based ELISA, immunofluorescence microscopy. The phage peptide bearing the 7-amino acid sequence, LGLRGSL, demonstrated selective binding to EMT phenotypic cells (MCF-7/TGF-β and MDA-MB-231) as compared to epithelial subtype, MCF-7, T47D and breast fibroblasts (Hs578T). The selected phage was also able to identify metastatic breast cancer tumor in breast cancer tissue microarray (TMA). These studies suggest that the selected phage peptide LGLRGSL identified by phage-display library, showed significant ability to bind to mesenchymal-like breast cancer cells/ tissues and can serve as a novel probe/ligand for metastatic breast cancer diagnostic and imaging.

## Introduction

Breast cancer is the most common cancer in women and the second leading causes of death due to cancer (1). The cause of death in breast cancer is often due metastasis to distant sites, resulting in organ failure accounting for a 5-year survival rate of 23%. Evidences support the observation that metastasis is an early event in breast cancer progression (2), with possibly up to 90% of patients already having metastasis at the time of diagnosis. Studies have shown that dissemination of cancer cells and metastasis into distant organs is often preceded by an epithelial to mesenchymal transition (EMT) of cancer cells (3), which allows cancer cells to dedifferentiate, acquire mesenchymal including fibroblast-like morphology, enhanced migratory and invasive properties, enabling them to invade through the stroma and migrate and seed to distant organs (4, 5). The concept of EMT in breast cancer has been well demonstrated in numerous *invitro* studies in different normal, malignant mammary epithelial cells and in mouse models of mammary cancers (6, 7). It has been suggested that tumor microenvironment (8) and growth factors such as transforming growth factor-β (TGFβ), epidermal growth factor (EGF), platelet-derived growth factor (PDGF) has a dramatic effect on epithelial phenotype and in promoting motility and invasiveness via the induction of EMT (9, 10). TGFβ treatment changes epithelial cells from cubodial shape to more elongated ones with concomitant loss of epithelial markers and increased expression of mesenchymal markers vimentin, fibronectin and α-smooth muscle actin (11). These EMT markers are also present in activated cancer-associated fibroblasts (CAF's), which contributes to the pathogenesis of tumor progression and invasiveness (12). Several studies support a physiologic role of EMT during tumor progression (13–15) by monitoring EMT progression by the cadherin switch, E-cadherin to N-cadherin, which is normally also present in mesenchymal cells, fibroblasts, neural tissue (16). Similarly, vimentin is also often used to define cancer cells undergoing EMT, is also present in fibroblasts, endothelial cells, cells of the hematopoietic lineages, and glial cells (17, 18). There is a lack of specific ligands that can recognize mesenchymal-like cancer cells and define EMT in tumor and in cancer-associated fibroblasts.

Phage display offers great advantage as a high throughput profiling technology based on peptide libraries present on the surface of bacteriophage. Selective binding of phages from a library with billions of diversified peptides can make a clear distinction between two morphological same but functionally different targets and thus offers a complementary approach for comparative screening. Usually peptides can be displayed on the N-terminus of pIII protein coat protein (pIII phage display), which is displayed at one end of the filamentous phage in 3–5 copies (19) or can be displayed on the N-terminus of all copies of pVIII major coat protein (20). Diversity of pIII or pVIII combinatorial phage library has been exploited extensively to explore the cell surface repertoire of various cancer cells such as colon (21), prostate (22, 23), pancreatic (24), breast (25, 26) and to select many cell surface or cell internalizing peptides. Some of these highly specific and high affinity ligands have been used as diagnostic (24), molecular and targeting agents (27–30). Additionally, lamba (T7) phage display has been used to identify vascular zip codes (31) and markers for angiogenesis (32). These studies and more define the power of using combinatorial phage display to identify molecular differences and interactive regions of the proteins without knowing the nature of interaction.

In this study, we propose a novel and innovative study to use phage display libraries for identification of phages that can specifically and selectively bind to the mesenchymal breast cancer cells *invitro*. Since TGFβ is a known inducer of EMT, we have used a model of TGFβ induced EMT in MCF-7 breast cancer cells, (MCF-7/TGFβ) for selection of EMT-specific phages. CX7C PhD phage library was used for selection of phages binding to MCF-7/TGFβ cells after subtractive depletion from breast fibroblasts. These selected phages were then tested on breast cancer cells that exhibited EMT phenotype (MDA-MB-231 and T47D-shNMI) and breast cancer TMA of primary and metastatic site. The phage peptide LGLRGSL displayed specific binding to the EMT breast cancer cells as well recognized tumor in TMA's at primary as well as metastatic site.

## Materials and Methods

### Materials

PhD CX7C phage library was purchased from New England Biolabs (NEB). Fetal calf serum (FCS) and cell culture media (Dulbecco's modified Eagle's medium, DMEM) was purchased from Sigma (USA). The phage display library contains random peptides constructed at the N terminus of the minor coat protein (cpIII) of M13 phage. The library contains a mixture of 3.1 × 10^9^ individual clones, representing repertoire of phages with 7-mer peptide sequences, which expresses random 7-amino-acid sequences. The *Escherichia coli* host strain ER2738 (F+ strain, New England Biolabs) was used for M13 phage propagation. The human breast cancer cell lines MDA-MB-231, MCF-7 and breast fibroblasts (Hs 578T) were purchased from the American Type Culture Collection. MCF-7 cells were treated 1ng/mL of TGFβ for 16 days. MCF-7, MDA-MB-231, MCF-7/TGFβ, breast cancer cells, and SW620, colon cancer cells, were maintained in DMEM supplemented with 10% fetal bovine serum (Sigma) at 37°C. PC3, prostate cancer cells, were cultured in RPMI1640 media supplemented with 10% FBS at 37°C.Breast fibroblasts (Hs578T) were maintained in special hybricare medium supplemented with 15% FBS (ATCC).

### Validation of EMT Marker in MCF7/TGFβ Cells by Western Blot

MCF-7 and MCF-7/TGFβ cells were grown in 25 cm^2^ flask to 75–80% confluency. Confluent cells were lysed in ice-cold complete 1x RIPA buffer (PMSF solution, sodium orthovanadate solution, protease inhibitor cocktail solution, and 1x lysis buffer) (Santa Cruz Biotechnology, Santa Cruz, CA, United States). The protein concentration in the samples was quantified using the BCA Protein Assay Kit (Pierce Biotechnology, Rockford, IL, United States). Thirty microgram of protein from each sample was separated by a 4–12% SDS-PAGE gel and then transferred to a 0.2 μm polyvinylidene difluoride (PVDF) membrane. Membranes were blocked with 5% nonfat dry milk in PBS-T for 45 min and then incubated with the E-cad herin (Abcam, UK) or N-cadherin (Abcam, UK) primary antibody (1:1,000) overnight at 4°C. After washing, membranes were incubated with horseradish peroxidase (HRP)-conjugated secondary antibody (1:2,000). Subsequently, membranes were washed and blots were visualized using enhanced chemiluminescence. The membrane was stripped with mild stripping buffer and reprobed with β-actin (Cell Signaling, Danvers, MA, United States) to verify that equal amount of protein was loaded. The relative quantification was normalized against β-actin using image J image analysis software.

### *In vitro* Phage Selection

Biased protocol for selection of phages was employed as described (26) with some modifications. The PhD phage library (Cx7C) was depleted against a cell culture flask and breast fibroblasts (Hs578T). Unbound phages recovered from the depletion were incubated with confluent MCF-7/TGFβ cells at room temperature for 1 h. Unbound phages were washed away and cell-associated phages were eluted with elution buffer (200 mM glycine-HCl, 1 mg/ml BSA, 0.1 mg/ml phenol red, pH 2.2) for 10 min on ice. The eluate was neutralized with 376 μl of 1 M Tris (pH 9.1). Internalized phages were recovered with lysis buffer [2% CHAPS, 10 mM Tris, 2 mM EDTA (pH 8.0)] after further washing and propagated in bacteria to determine their titer as described previously (29). The results were expressed as a percentage of a ratio of output to input phage. The eluted phage and cell-internalized phage were amplified separately in bacteria and used in the second and third round of selection using the same protocol of depletion of the amplified phages (lysate and eluate) against breast fibroblasts and incubating MCF-7/TGFβ cells with unbound phages recovered from depletion. Sixty phages from the third round of selection were randomly picked and were propagated in the ER2738 bacteria. DNA was isolated form these 60 propagated clones using DNA isolation kit (QIAGEN GmbH, Hilden, Germany) and individual phage DNA sequences were identified. A sequencing primer used was 5′-CCC TCA TAG TTA GCG TAA CG-3′ (−96 gIII sequencing primer, provided in the Ph.D.-CX7C Phage display peptide library kit (NEB, MA).

### Cell-Based ELISA and Phage Capture Assay

Selected phage clones were characterized for their selectivity toward EMT cells, MCF-7/TGFβ and MDA-MB-231 breast cancer cells in comparison with epithelial breast cancer cells, MCF-7, T47D, and breast fibroblasts using phage capture assay (29) and cell-based ELISA.

Briefly, in phage capture assay, target cells MCF-7/TGFβ, MDA-MB-231, MCF-7, T47D, T47D-shNMI, breast fibroblasts (Hs578T), PC3 (metastatic prostate cancer cells) and SW620 (metastatic colon cancer cells) were cultured in triplicate to confluence in separate wells of 12-well cell culture plates. Cells were incubated with phage (1 × 10^10^ pfu) at RT for 1.5 h. Cells were washed with 100 μl washing buffer for 5 min eight times to remove non-specifically interacting unbound phages. Cells were lysed with 50 μl lysis buffer (2.5% CHAPS) for 10 min on a rocker and the lysate containing phages was titered in *E. coli* ER2738 bacterial cells. Phage titer was calculated as a ratio of output to input phage.

#### ELISA:

Confluent monolayers of MCF-7/TGFβ, MDA-MB-231, MCF-7, T47D, T47D-shNMI and breast fibroblasts (Hs578T) cells were incubated at room temperature with individual phage clones (10^10^ PFU), for 1.5 h at RT. Subsequently, cells were washed with PBS containing 0.1% Tween-20, incubated with primary anti-M13-biotin antibody (1:1,000), for 1 h, at RT. Cells were washed again with PBS containing 0.1% Tween-20, incubated with secondary antibody streptavidin-HRP (1:2,000, 45 min, RT), developed with tetra methyl benzidine and read at absorbance 650 with microplate reader (BioTek).

### Phage Capture Assay of Phage Binding to Cancer-Conditioned Media Activated Fibroblasts

Breast fibroblasts (Hs578T) were plated in a 12.5 cm^2^ flask cultured until approximately 70% confluent. Once properly confluent, fibroblasts were then cultured in MDA-MB-231 conditioned media or normal fibroblasts media for 72 h. Thereafter, they were exposed to E11 phage (10^8^ pfu) for 2 h and analyzed for binding in phage capture assay as described above.

### Immunofluorescence Study of Selected Phages

MCF-7, MCF-7/TGFβ, MDA-MB-231 and Hs578T (breast fibroblasts) cells were seeded in 4-well chamber overnight. On next day, cells were fed with fresh medium. Phage LGLRGSL (E11) (10^8^ pfu) was added in fresh medium and incubated at RT for 1 h. After removing the unbound phages, cells were washed with wash buffer (0.1% tween-20 in PBS) three times and fixed with 4% formaldehyde for 15 min at 37°C. Thereafter, cells were permeabilized with 0.2% Triton X-100 at RT for 10 min. Then, cells were washed with TBS 3 times. Before incubation with anti-phage antibody, cells were treated with blocking buffer for 30 min at RT. Cells were incubated with M13-pIII monoclonal antibody for 1 h at RT, washed and incubated with the secondary goat anti-mouse IgG antibody labeled with Alexa Flour® 488 (Molecular Probes) (1:500 in PBS containing 1% BSA) for 45 min at RT. Subsequently, cells were washed three times and stained with TOTO-3 for nucleus staining. Prolong Gold Anti-fade Reagents was used on the cells which were then covered with cover slides. Pictures were taken by using the NIKON eclipse TE 2000-E confocal microscope. The fluorescence intensity of the images was quantified using image J software.

### Phage Binding to Breast Cancer Tissue Microarrays

The breast tissue microarrays were purchased from Novus Biological (Littleton, CO). TMA included 40 breast cancer infiltrating ductal carcinoma, 10 metastatic lymph node and 9 adjacent normal breast tissues. Clinico-histopathologic characteristics of the subjects in the tissue microarray study included grade, age, hormone status and clinical stage, according to information provided by the suppliers. Tissues were de-paraffinized in xylene, rehydrated in graded alcohols and endogenous peroxidase activity was quenched with 3% hydrogen peroxide for 5 min. Slides were treated with LGLRGSL phage (10^10^ pfu) overnight. Slides were subsequently washed and blocked by 3% goat serum at RT for 1 h in humidity chambers. Slides were then treated with M13-pIII phage monoclonal antibody (NEB, MA) or Vimentin antibody (Cell Siganling, Danvers, MA, United States) (1:100) and then subsequently with HRP conjugated goat anti-mouse secondary antibody (Jackson Immunoresearch Laboratories Inc., West Grove, PA, United States) for 40 min. The antigen-antibody reaction was visualized after applying diaminobenzidine (Sigma-Aldrich, MO, United States) for 7 min. The slides were counterstained with hematoxylin (Sigma-Aldrich, MO, United States) for 1 min. Slides were dehydrated in alcohols and cleared in xylene baths before being mounted with Permount media.

### Statistics

The significance of difference between two variables was assessed by the Student's *t*-test. The difference was considered significant if the *p*-value was <0.05. Data from all experiments are expressed as mean ± standard error (SD). All statistical calculations were performed using GraphPad Prism and Microsoft Excel.

## Results

### Selection of Phages Binding to Breast Cancer Cells That Have Undergone EMT

**Figure 1:**
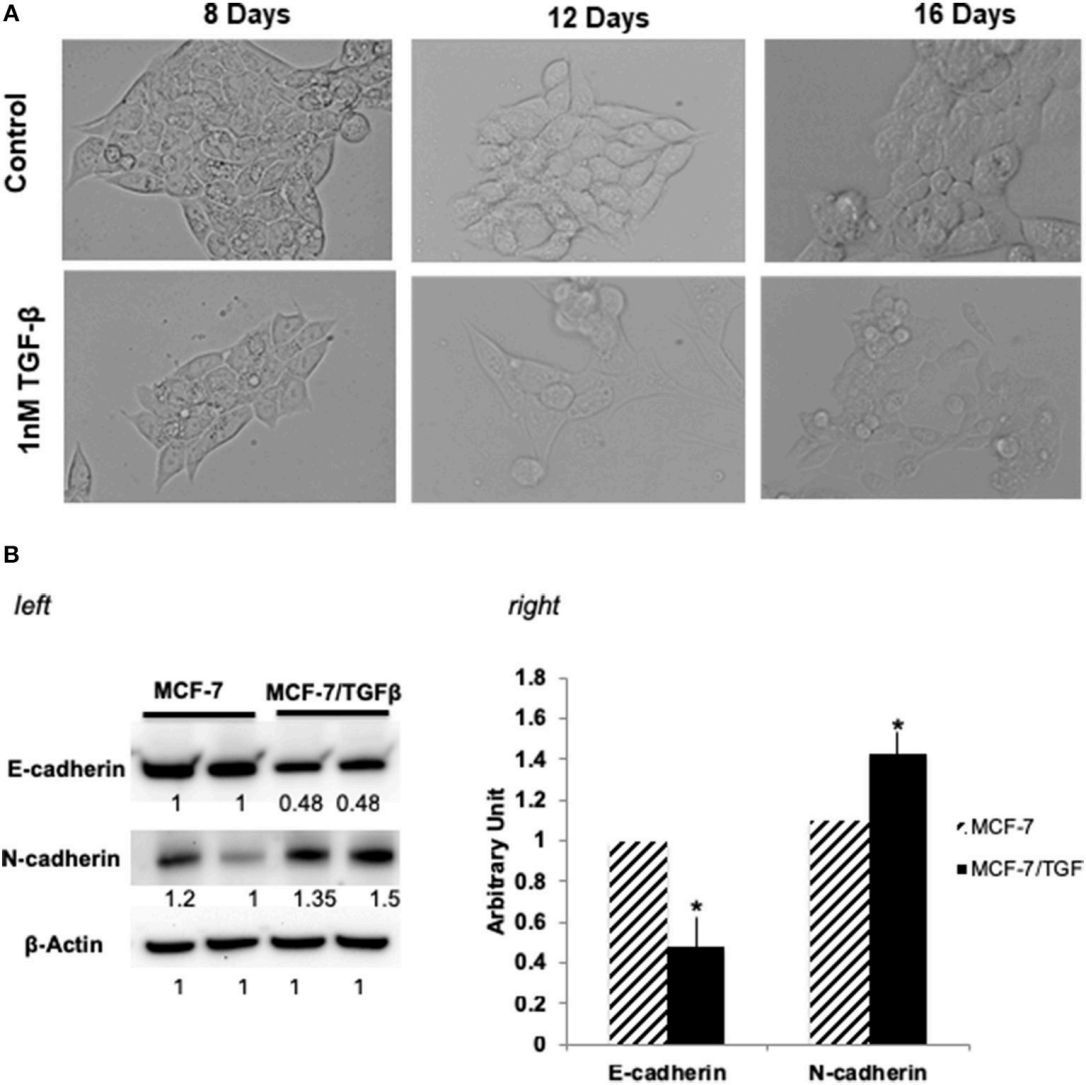
**(A)** Morphological change in MCF-7 cells during TGFβ –induced EMT. Images of cells treated long term (16 days) with TGFβ showing spindle-shaped morphology as compared with control. Images were acquired by phase contrast microscopy using a 20 × objective. **(B) (left)** Immunoblot analysis of expression of EMT-related proteins. Protein expression levels of E-cadherin in TGFβ-treated MCF-7 cells were markedly decreased, whereas expression levels of N-cadherin and vimentin (mesenchymal markers) were dramatically increased. Numbers below each panel indicate the relative integrated density of the protein band in that lane. **(right)** Quantification analysis of the Western blot data showing the change in EMT markers (E-cadherin and N- cadherin) in MCF-7/TGFβ cells vs. MCF-7 breast cancer cells. The relative quantification was normalized against β-actin using image J image analysis software. All data represent the mean± S.D of three different experiments. ^*^p <0.05, student-*t*-test.

MCF-7 (epithelial-luminal subtype) breast cancer cells were transformed into mesenchymal phenotype by long-term treatment with TGFβ (1 ng/mL for 16 days). Figure 1A shows the change of MCF-7 breast cancer cells change in morphology upon TGFβ treatment. Since reduction in E-cadherin and upregulation of mesenchyme markers, is a hallmark of metasatatic carcinoma's and indication of EMT (33, 34), following treatment MCF-7/TGFβ cells were validated for EMT transition by looking at the protein expression of E-cadherin and N-cadherin (mesenchymal marker) (35). Consistent with literature that (33) demonstrated that TGFβ treatment downregulates E-cadherin expression in MCF-7 cells, our Western blot data confirmed these observations. Figure 1B showed downregulation of E-cadherin and upregulation of N-cadherin protein expression in MCF-7/TGFβ cells as compared to MCF-7 cells.

**Figure 2:**
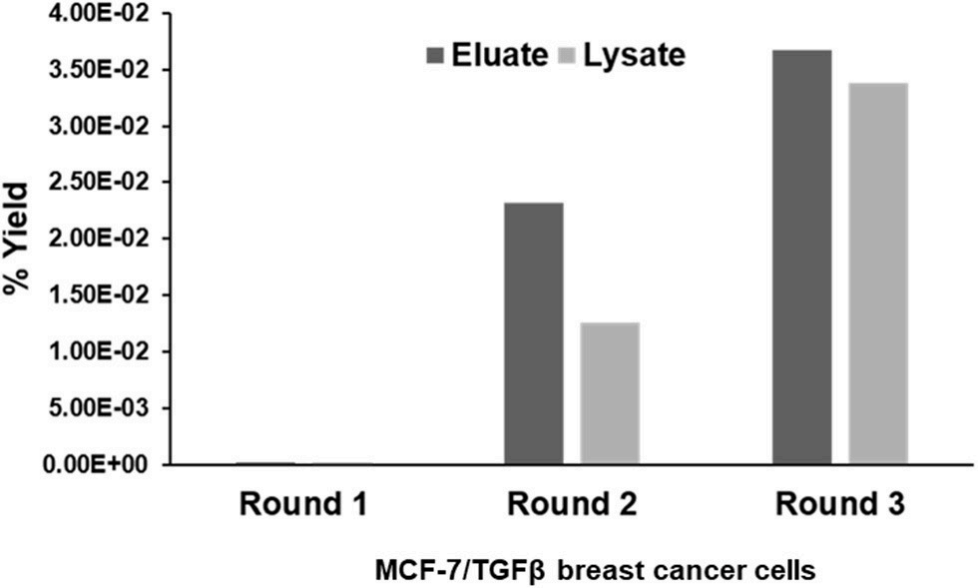
Specific enrichment of eluate and lysate MCF-7/TGFβ cell-binding phage isolated from PhD CX7C library during three rounds of selection. The titer of recovered phages from each round was evaluated by blue plaque-forming assay on agar plates. The phage enrichment rate was calculated as yield (%), which is as output number/input number x100.

CX7C PhD phage library (NEB) was used to find phage clones that bind with high specificity and selectivity to MCF-7/TGFβ cells. Extensive depletion of the phage library against plastic, breast fibroblasts before enrichment of phage that interact with MCF-7-TGFβ breast cancer cells was employed for a robust selection of phage clones specific for cancer cells. This negative selection step was also performed after each round of panning on the MCF-7-TGFβ cells. Three such rounds of biopanning were performed on and in every round, phage library and sub-library was depleted against breast fibroblasts to preferentially select for phages that did not bind to normal fibroblasts. Phages associated with cells were eluted sequentially with acid and detergents. Titer of the phage increased from one round to another indicating successful enrichment for phage clones that bind to the target MCF-7-TGFβ cells (Figure 2). After the third round of selection, 100 phage clones were randomly picked after titering of the eluate and lysate fractions. Their DNA was isolated, sequenced and translated to reveal the sequence of the pIII fusion peptides. In total, 21 phage clones were isolated and classified based on their consensus foreign peptide motifs (Table 1).

**Table 1:** Displayed phage peptide sequences from isolated eluate and lysate phages from third round of selection against MCF/TGFβ breast cancer cells.

**ELUATE PHAGE PEPTIDE SEQUENCES**							
E9	I	L	N	C	M	R	N
E11	L	G	L	R	G	S	L
E12	A	R	K	T	N	P	L
E16	F	N	G	P	H	T	R
E20	T	K	F	H	F	S	G
E25	D	F	L	T	A	R	L
E29	N	T	F	S	W	H	T
E32	G	T	F	L	F	S	
E42	N	T	L	R	T	P	Y
E43	H	H	D	N	V	A	M
E45	P	N	L	P	W	V	P
E46	Y	E	H	H	P	R	I
E48	H	M	R	Q	G	M	A
**LYSATE PHAGE PEPTIDE SEQUENCES**							
L5	T	H	S	S	W	G	M
L9	N	M	W	E	S	V	P
L10	R	E	G	H	M	G	V
L24	K	D	S	H	E	P	W
L27	T	L	A	T	G	G	M
L30	P	Y	E	P	R	A	T
L42	K	G	D	Y	K	L	F
L45	S	I	L	S	K	N	H
L46	E	R	S	G	M	H	S
L47	H	W	P	A	K	H	I
L49	P	V	L	L	G	E	S

### Selectivity of Phages Toward Mesenchymal-Like Breast Cancer Cells

**Figure 3:**
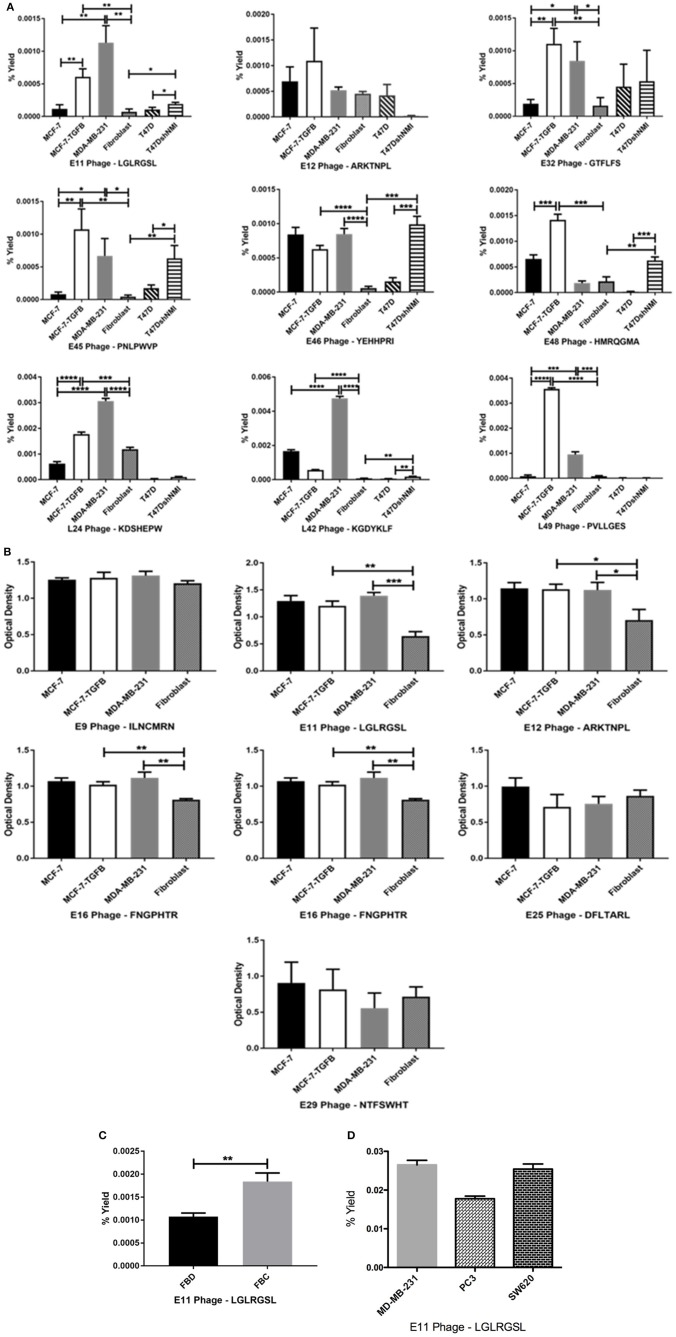
Affinity selected eluate and lysate phage showed higher binding to MCF-7-TGFβ, MDA-MB-231, T47D-shNMI cells as compared to breast fibroblasts, T47D and MCF-7 cells in **(A)** phage capture assay; **(B)** in phage based-ELISA; **(C)** LGLRGSL (E11) was also highly reactive to activated fibroblasts. FBD denotes fibroblasts in normal fibroblast media and FBC denotes fibroblasts in MDA-MB-231 breast cancer cell conditioned media and **(D)** LGLRGSL showed comparable binding to PC3 and SW620 cancer cells as compared to MDA-MB-231 cancer cells. All data represent the mean± S.D. ^*^p <0.05, ^**^p ≤ 0.01, ^***^p ≤ 0.001, and ^****^p ≤ 0.0001.

Phage clones obtained by screening of the CX7C phage library against MCF-7/TGFβ cancer cells were tested for their selective binding toward the target MCF-7/TGFβ, MDA-MB-231, T47D-shNMI cells and not to breast fibroblasts or epithelial subtype breast cancer cells MCF-7 and T47D in phage capture assay (Figures 3A,B) and phage based ELISA (Figure 3C).

These cells lines MCF-7/TGFβ, MDA-MB-231, T47D-NMI exhibit mesenchymal phenotype or markers of EMT and are aggressive, are structurally similar to fibroblasts and expresses markers of EMT and thus are representation of EMT in breast cancer cells. MDA-MB-231 breast cancer cell line exhibit mesenchymal phenotype and are detonated EMT phenotype (36). T47D is an epithelial breast cancer cell line and was transitioned to EMT by silencing a gene, N-myc and STAT interactor (37).

In these assays, some phages demonstrated high selectivity toward EMT cells, while other phage showed selectivity for epithelial breast cancer cells as well as breast fibroblasts. Phages were considered selective if their relative binding to EMT phenotypic cells (MCF-7/TGFβ, MDA-MB-231, and T47D-shNMI) and were at least five times higher than those of epithelial breast cancer cells (MCF-7 and T47D) and breast fibroblasts. KGDYKLF (L42), phage selected from lysate fraction, showed high specificity toward MDA-MB-231 cells but not so selective toward MCF-7/TGFβ, MCF-7 and breast fibroblasts. Phages selected from eluate fraction, LGLRGSL (E11), GTFLFS (E32), and PNLPWVP (E45) were very selective for EMT phenotypic cells (MCF-7/TGFβ, MDA-MB-231, and T47D-shNMI) and showed more than 10 times binding as compared to its binding to breast fibroblasts (Hs578T) and epithelial breast cancer cells (MCF-7 and T47D) in phage capture assay (Figure 3A). Phage E11 was confirmatory toward EMT cells in phage-based ELISA (Figure 3B) and thus was chosen for further characterization.

To determine if E11 could recognize EMT phenotype in other cell types of tumor microenvironment, E11 was screened against activated fibroblasts (fibroblasts converted to CAF's by treatment with cancer-conditioned media). E11 demonstrated higher binding (twice as much) to activated-fibroblasts than normal fibroblasts (Figure 3C). To see if E11 can recognize EMT on cancer other than breast, E11 was screened against other metastatic cancer cells, PC3 (prostate cancer) and SW620 (colon cancer) in phage capture assay. PC3 is a highly metastatic prostate cancer cell line and exhibits EMT phenotype (38, 39). SW620 are highly tumourigenic, metastatic and exhibit fibroblasts like morphology (40). E11 showed comparable binding to PC3 and SW620 like MDA-MB-231 (Figure 3D), which demonstrates that it is binding to a receptor common to metastatic phenotype.

### Affirmation of Phages Binding to Target Cells *in vitro* Using Immunofluoresence Analysis

**Figure 4:**
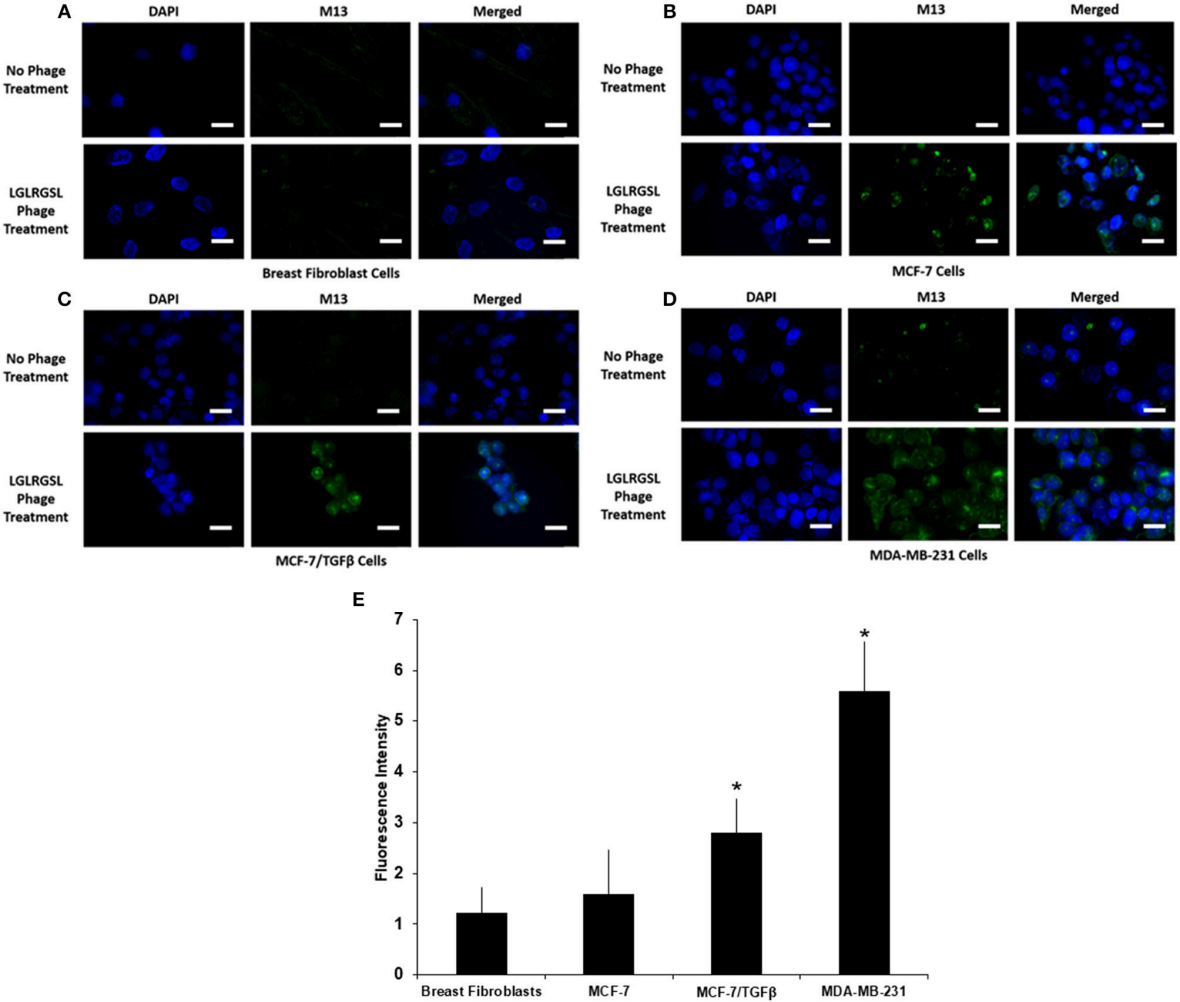
A. Phage peptide LGLRGSL (E11) stained selective to EMT phenotypic cells in immunofluorescence microscopy analysis upper, **(A)** Breast Fibroblast, **(B)** MCF-7, **(C)** MCF-7/TGFβ, and **(D)** MDA-MB-231 cells without phage exposure; stained with DAPI and Alexa 488 secondary antibody, lower, **(A)** Breast Fibroblasts, **(B)**MCF-7, **(C)** MCF-7/TGF β, and **(D)**. MDA-MB-231 exposed to phage; stained with M13 primary antibody, DAPI and Alexa 488 secondary antibody. Scale bar is 20 μm. **(E)** Quantification data of the fluorescence intensity of the Breast fibroblasts, MCF-7, MCF-7/TGFβ, and MDA-MB-231 breast cancer cells. The fluorescence intensity of the images was quantified using Image J software. All data represent the mean± S.D of three different experiments. ^*^p <0.05, student-*t*-test.

To further affirm the specificity of LGLRGSL (E11) toward breast cancer cells with an EMT phenotype, immunofluorescence microscopy of intact target mesenchymal phenotypic cells (MCF-7/TGF β and MDA-MB-231), control MCF-7 breast cancer cells and breast fibroblasts (Hs578T) was employed. All cells were treated with the phage (10^8^ pfu) at RT for 1 h, and subsequently incubated with primary anti-pIII antibody and then stained with secondary anti-mouse Alexa fluor 488 secondary antibody. LGLRGSL (E11) showed almost no binding to breast fibroblasts (Figure 4A), some staining to MCF-7 cells (Figure 4B), while abundant binding to EMT cells, MCF-7/TGFβ (Figure 4C) and MDA-MB-231 (Figure 4D) as shown by green fluorescent phage staining and analysis (Figure 4E). We did not observe any background antibody as shown in the respective controls of cells treated with just primary and secondary antibodies.

### Validation of Phage Peptide Binding to Human Breast Cancer *Exvivo*

**Figure 5:**
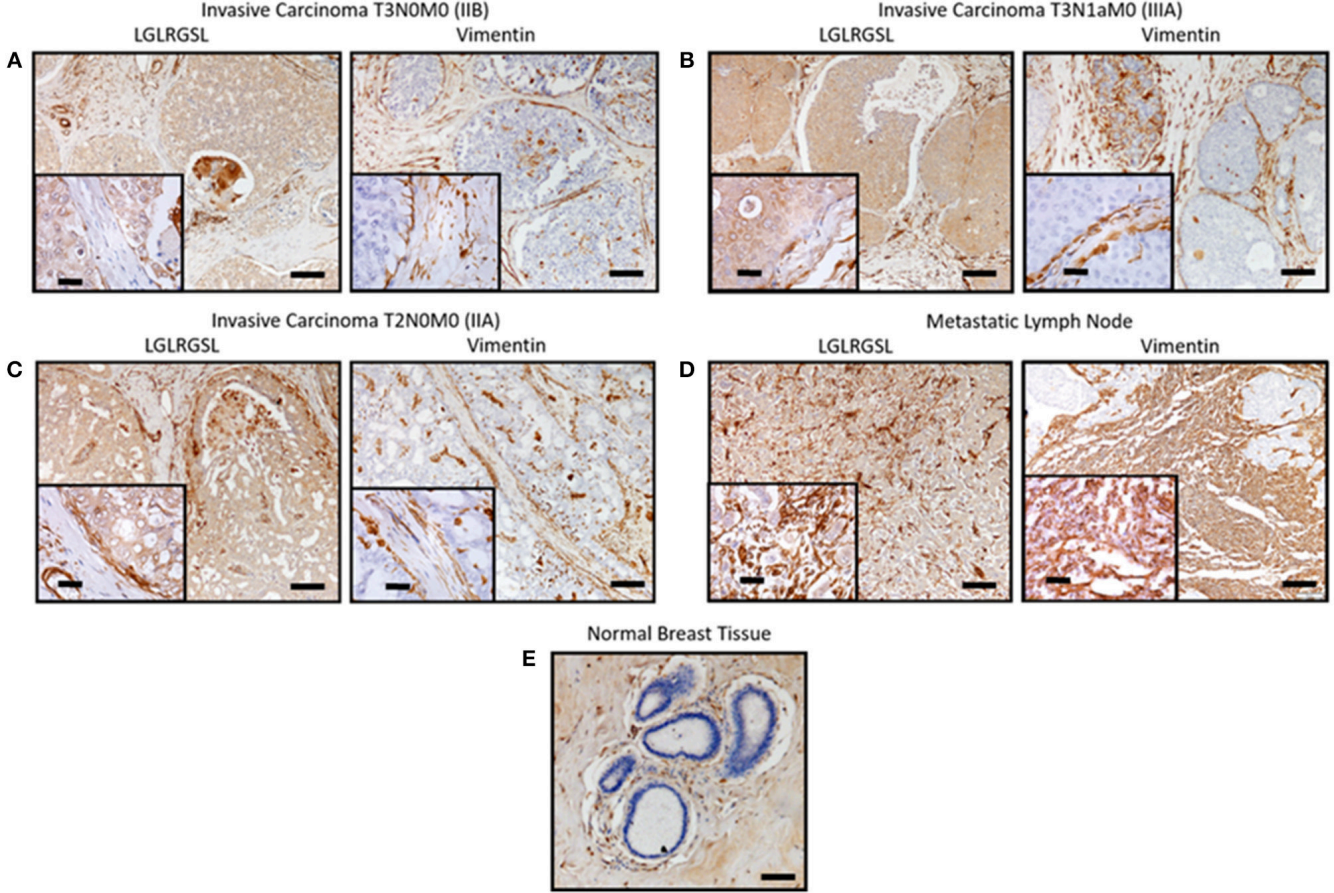
*Ex vivo* phage binding to human breast cancer tissue microarray. Tissue microarray of invasive ductal carcinoma and adjacent normal tissues were incubated with 10^10^ pfu of LGLRGSL phage or Vimentin antibody and then subsequently with M13-pIII antibody for phage and secondary peroxidase antibody for phage and Vimentin, imaged with a digital light microscope. Strong brown staining of the phage and Vimentin was observed in invasive ductal carcinoma sections **(A–C)** and metastasis in lymph nodes, **(D)** while no staining was observed in normal breast tissue, **(E)** Scale Bar is 40 and 10 μm for the inserts.

Next, we investigated the clinical relevance of these findings by assessing if LGLRGSL (E11) could be used to prospectively identify human invasive ductal carcinoma (IDC) breast tumors with a propensity to metastasize as metastatic cells undergo EMT before metastasizing (41). Immunostaining for phage in human breast cancer tissue indicated phage has substantial staining for invasive ductal breast cancer carcinoma (Figures 5A–C, left) and its staining intensity increased in tumors invading into adjacent lymph nodes (Figure 5D). Furthermore, we did not observe any binding in normal breast tissues (Figure 5E). Interestingly we observed that vimentin, a mesenchymal marker, within the same TMA (Figures 5A–C, right) demonstrated a different staining pattern than the LGLRGSL (E11) phage. While vimentin showed stromal staining, phage was immunoreactive to the tumor cells with robust staining around the invasive or leading edge of the tumor-stromal interaction.

## Discussion

There is accumulating evidence to show that epithelial cells can undergo transformation into migratory fibroblast-like mesenchymal cells in a process called EMT (Epithelial-to-Mesenchymal Transition). Normally, an embryo and organ development related phenomenon, EMT is believed to be crucial for primary tumors to escape their original residence and invade and metastasize to other organs such as liver, lungs, bone and brain (42). Moreover, EMT is also a critical determinant of stemness and drug-related relapse (6, 41, 43). EMT of breast cancer cells is, in large part, dependent oncontingent on the tumor microenvironment (44). Because of the close cross-talk between the cancer cells and CAFs, it is evident that the development of cancer cannot be dissociated from its local microenvironment (45). Tumor cells signals stromal fibroblast cells and activate them into cancer-associated fibroblasts (CAFs) to undergo EMT through the stimulation of paracrine growth factors (46, 47) promotes EMT, cell survival (48) and progression (49) of cancer cells. To better understand the events involved from acquiring motility for invasion to seeding in distant organs, there is a need to develop probes that can selectively bind to invasive, metastatic and tumor-progressing CAF's (46). Such ligands can further ascertain the role of EMT in cancer metastasis and could enable the development of new approaches in the management of this disease.

In this study, we have successfully isolated phage ligands using CX7C phage library for EMT transformed breast cancer cells, MCF7/TGFβ and MDA-MB-231 by employing subtractive depletion of phages binding to breast fibroblasts. The optimizing procedures (several rounds of subtractive screening) were performed to improve the probability of successful selection, which is highly dependent on obtaining specific phages with high selectivity. The isolated clones were used in cell-ELISA and *invitro* phage capture assay to confirm their specificity to EMT phenotype cells, MCF-7/TGFβ, MDA-MB-231 and T47D-shNMI cells *in vitro* as compared to epithelial subtype cells, MCF-7, T47D and mesenchymal breast fibroblasts (Hs578T). Phage capture assay and ELISA demonstrated the selective affinity of various phages to EMT phenotype.

The best candidate, LGLRGSL (E11), was then selected for immunocytochemical assays. Immunofluorescence studies confirmed the selectivity of LGLRGSL (E11) to the target mesenchymal-like cells as there was minimal binding to the non-target epithelial breast cancer cells and mesenchymal breast fibroblasts. E11 also bound with great affinity to PC3, prostate cancer cells and SW620, colon cancer cells. It's binding to these other cancer cell type was as comparable as to MDA-MB-231 breast cancer cells. These findings suggest that LGLRGSL (E11) is recognizing a receptor/antigen on mesenchymal-like cancer cells that are highly invasive and metastatic in nature and would be a useful probe to identify invasive front and metastatic tumor cells. Phage probing to the breast cancer tissue microarray identified tumor representing high grade and lymph node metastasis. When compared to Vimentin, a marker of mesenchymal-like cells metastasis, phage had more positive staining to the invasive front and lymph node metastasis.

More work is needed to characterize LGLRGSL (E11) as ligand binding to EMT marker of cancer origin. One such direction is the identification of the receptors responsible for LGLRGSL (E11) phage binding to the mesenchymal-like cells, that may allow for the discovery of novel cell surface molecules, which may yield future targets for drug design.

In conclusion, the 7-amino acid phage peptide, LGLRGSL, obtained by phage-display technology showed significant ability to bind to EMT breast cancer cells *in vitro* and tissues array *exvivo*. The phage peptide can be used for preparation of targeted devices for drug and gene delivery to metastatic cells; development of probes for molecular imaging of metastasis; and identification and isolation of cancer-specific receptors as potential components for development of therapeutic antibodies, anticancer vaccines and diagnostics.

## Author Contributions

KJ study design, performed experiments, data analysis and interpretation, and manuscript preparation. KJ, BK, JJ-T, HH and MS: performed experiments and data analysis. RS, ST, CY, and DB project supervision, study design, and manuscript preparation.

### Conflict of Interest Statement

The authors declare that the research was conducted in the absence of any commercial or financial relationships that could be construed as a potential conflict of interest.
